# Isolated skeletal muscle recurrence of an originally nodal diffuse large B cell lymphoma: A case report and review of the literature

**DOI:** 10.1097/MD.0000000000009608

**Published:** 2018-01-19

**Authors:** Nikolaos Spetsieris, Nefeli Giannakopoulou, Eleni Variami, Konstantinos Zervakis, Niki Rougala, Georgios Garefalakis, Vasiliki Skarlatou, Nora-Athina Viniou, Panagiotis Diamantopoulos

**Affiliations:** National and Kapodistrian University of Athens, Laikon General Hospital, 1st Department of Internal Medicine, Haematology Unit, Athens, Greece.

**Keywords:** extranodal recurrence, lymphoma relapse, lymphoma relapse radiographic images, lymphoma second-line treatment, muscle infiltration

## Abstract

**Rationale:**

Diffuse large B cell lymphoma (DLBCL) is a malignancy of the B cells with extranodal primary involvement being estimated at 30% to 40% of cases. Primary skeletal muscle presentation of DLBCL is extremely rare, with an estimated incidence of about 0.5% of extranodal lymphomas, presenting mostly in the lower extremities. The possible mechanisms of muscle involvement of DLBCL include primary extranodal disease, extension from adjacent organs (such as lymph nodes) or disseminated disease.

**Patient concerns:**

We report a case of a 70-year-old woman with an advanced initially nodal DLBCL, treated with R-CHOP, that presented with an enlargement of her left thigh and restricted mobility 3 months after completion of chemotherapy. Imaging studies were performed, which showed possible infiltration of the muscles of the left thigh, without any nodal disease present.

**Diagnoses:**

Muscle biopsy documented the recurrence of the lymphoma at the left thigh.

**Interventions:**

The patient started second-line treatment with gemcitabine and vinorelbine.

**Outcomes:**

A partial response was achieved after the first cycle.

**Lessons:**

The remarkable element lies in the reappearance of the lymphoma at the left thigh muscles, with no radiographic or clinical evidence of involvement of lymph nodes, despite the extensive lymph node disease at initial presentation. The further management of such recurrences remains to be clarified, as the odd biological behavior of the malignant cells dictates a special handling of the disease.

## 1 Introduction

Diffuse large B cell lymphoma (DLBCL) is a malignancy of the B cells with a significant clinical, morphological, and molecular heterogeneity. It is the most common lymphoid malignancy in adults, accounting for 30% to 40% of all non-Hodgkin lymphomas (NHLs) in western countries.^[1]^ The median age of occurrence is in the seventh decade, and symptoms at presentation, behavior, and prognosis are dependent on the type and the primary site of the lymphoma. The malignancy typically occurs in the lymph nodes, with extranodal primary involvement being estimated at 30% to 40% of cases.^[2,3]^ The gastrointestinal tract is mostly affected, while extranodal sites of involvement described include the testes, skin, lung, bone, central nervous system, and the respiratory system.^[2,3]^ Primary skeletal muscle presentation of DLBCL is extremely rare, with an estimated incidence of about 0,5% of extranodal lymphomas,^[3]^ presenting mostly in the lower extremities.^[2]^ Involvement of the skeletal muscles upon recurrence of an initially nodal DLBCL is rare. The possible mechanisms of muscle involvement of DLBCL include primary extranodal disease, extension from adjacent organs (such as lymph nodes) or disseminated disease.^[3]^ The case presented here falls in the latter category, as our patient had an initially nodal DLBCL that recurred in the muscles of the left thigh.

## 2 Case presentation

A 70-year-old woman presented to the Internal Medicine Department due to a rapidly enlarging mass in the left submandibular region. The patient was a nonsmoker and had a medical history of diabetes mellitus type 2, arterial hypertension, and heterozygous beta thalassemia. She was under treatment with nebivolol, metformin, repaglinide, and vildagliptin.

The general clinical examination was unremarkable except for 2 enlarged, palpable submandibular and cervical lymph nodes. The submandibular lymph node infiltrated the adjacent bone structure of the jaw.

A biopsy of the submandibular lesion showed an aggressive B- lymphoma, with features compatible with an Epstein–Barr virus (EBV) (-) DLBCL non-otherwise specified (DLBCL-NOS, WHO 2008), with immunomorphological features of postgerminal center activated B cells [ABC, CD20+, CD79a+, PAX-5+. CD30+, MUM-1+, bcl-6+, MYC < 40%, EMA+, bcl-2+, Sig/Cig(λ)+, CD10−, CD15−, CD56−, CD138−, LANA-1-]. Large cells resembling immunoblasts as well as plasmablasts and centroblasts (in a smaller proportion) were observed. Bone marrow biopsy documented a substantially limited (<2%) infiltration from a small B-cell population, which did not provide evidence of invasion in the context of minimal disease. The possibility of molecular disease could not be excluded.

The patient was staged as having stage III disease, according to the Ann Arbor Staging system (enlarged lymph nodes in both axillary cavities, enlarged subcarinal lymph nodes, beside the superior vena cava, in the aortopulmonary window and the posterior mediastinum, enlarged paraortic lymph nodes beside the superior pole of both kidneys, along the psoas muscles, on the left and right iliac chain and along both femoroinguinal regions) and was started treatment with rituximab, cyclophosphamide, vincristine, doxorubicin, and prednisone (R-CHOP). After 6 cycles of chemotherapy, the patient had shown a complete response to the treatment.

**Figure 1:**
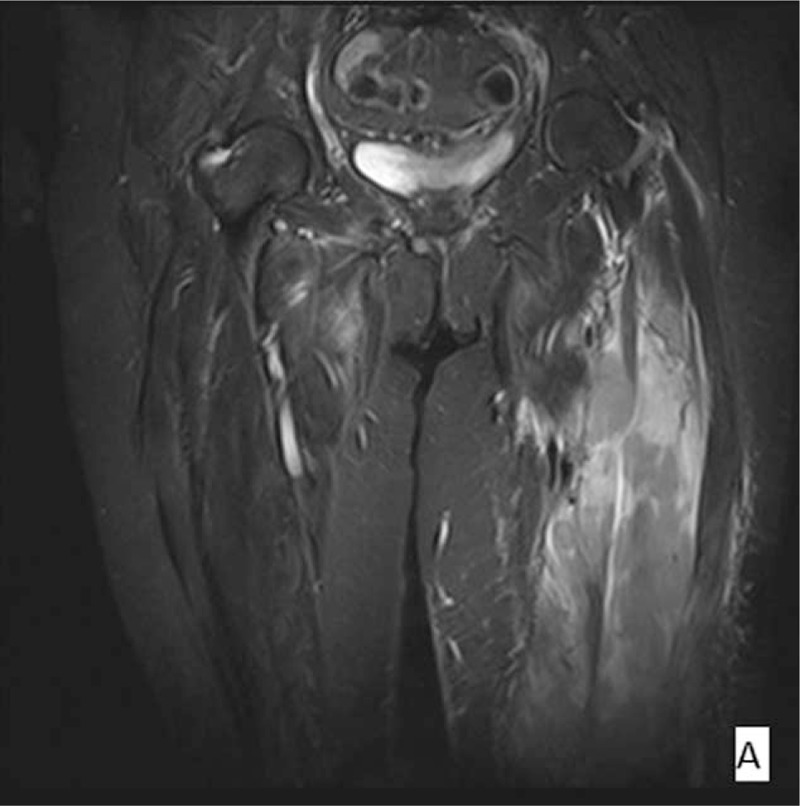
Coronal fat-suppressed T2-weighted image shows an infiltrating soft tissue mass of high signal intensity involving the anterior and lateral compartment of left femur and the internal adductor muscles of the right femur. Subcutaneous stranding is present.^[4]^

**Figure 2:**
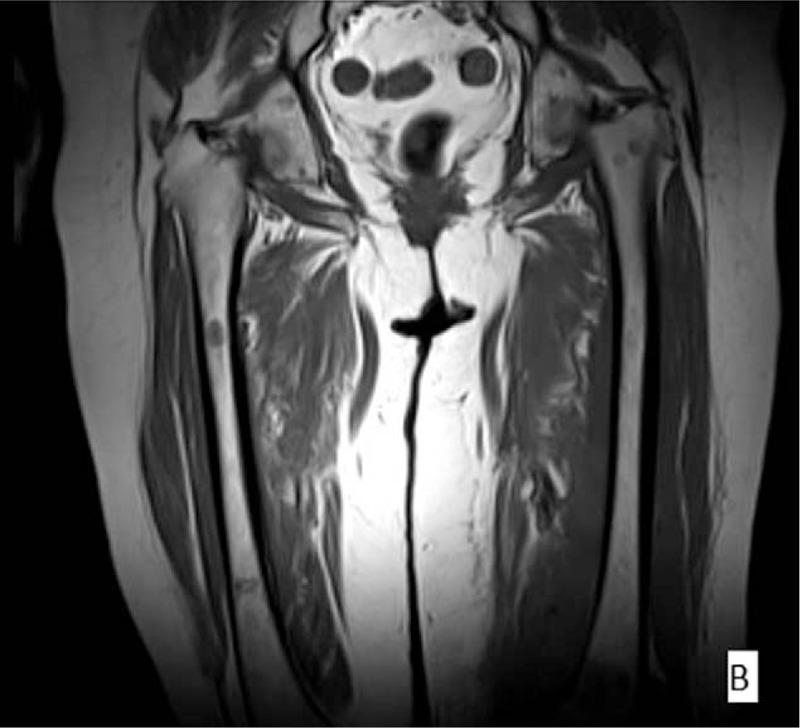
Coronal T1-weighted image.^[4]^

**Figure 3:**
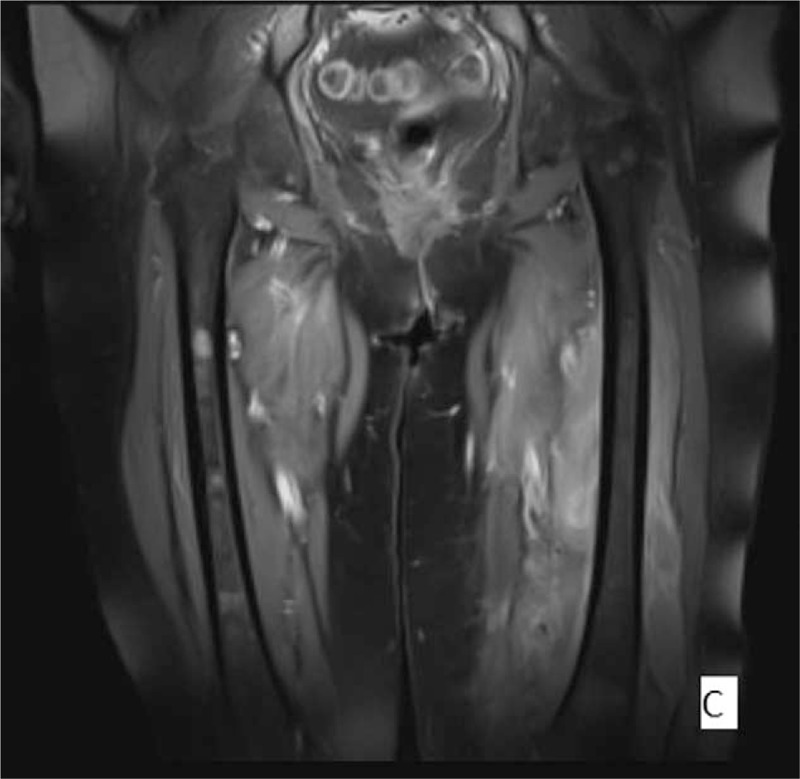
Left- suppressed contrast enhanced T1-weighted image (Image 3) and Image 2 show multiple foci of T1 hypointense and enhancing marrow lesions in both femora and extensive multicompartmental soft tissue mass involvement with peripheral thick band-like enhancement.^[4]^

Three months after the completion of the initial treatment regimen, the patient presented with swelling, redness, and tenderness on her left thigh, which restricted the leg's mobility. Ultrasonography of the lesion described a hypoechoic region with increased vascularity below the subcutaneous fat in the internal surface of the thigh. Computed tomographic (CT) scan revealed an enlargement of the muscles of the left thigh, as well as an effusion between the muscles and in the knee joint. Magnetic resonance imaging (MRI) findings included architectural distortion of the left adductor magnus, the intermediate and lateral vastus muscle, and mainly the biceps femoris, with significant pathologic magnetic signal and enhancement by the MR contrast material. Similar lesions, of lesser extent, were observed at the right adductor magnus, without contrast enhancement (Figs. 1–3).^[4]^ A venous occlusion was ruled out, with 2 lower limb venous ultrasonography examinations that were performed at different times. The lesion was biopsied with CT guidance. The histology reported infiltration of skeletal muscle fibers by a large B-cell neoplastic population, positive for CD30, with a Ki67 proliferation rate of 90%. A bone marrow biopsy showed no malignant infiltration. Apart from the skeletal muscle site, no other site of recurrence was documented.

The patient was started with gemcitabine and vinorelbine, achieving a partial response after the first cycle. The leg's full mobility was not restored, but the mass has significantly decreased in size. At present, she is still under palliative chemotherapy.

## 3 Discussion

The most common presentation symptoms of DLBCL are B-symptoms, including fever, night sweats, and significant weight loss. The patient may also present with anorexia, fatigue, chest or abdominal discomfort, shortness of breath, and neurological manifestations. Upon physical examination, the findings differ based on the possible extranodal site of the lymphoma, its relationship to adjacent organs or systems, the lymphoma subtype, stage, and the tumor burden. Common findings include lymphadenopathy (cervical, axillary, inguinal), splenomegaly, fever, and edema of the extremities. Skeletal muscle invasion may cause muscle swelling, pain, and edema, without redness or heat sensation.^[3]^ In other cases, it may present with skin and soft tissue changes suggesting cellulitis. A rapidly enlarging mass may be palpated. Furthermore, the infiltration of muscles may cause neurological manifestations due to nerve involvement, such as piriformis syndrome or cranial peripheral nerve palsy.^[2]^ Cases with facial muscle and mastication muscle infiltration have been described, with the patient presenting with facial hemiplegia, paresthesia, and swelling. Other uncommon manifestations described are that of an acute compartment syndrome, due to the compression of vascular structures, and a perianal abscess-like presentation. The most common site of involvement is the extremities, with psoas, calf, gluteal, triceps muscle invasion being described in the literature.^[2,3,5,6]^

Radiological assessment and staging of the disease is performed by ultrasound, radiographs, CT, MRI, and positron emission tomography (PET)/CT. In musculoskeletal involvement, radiographic images may show only soft tissue alterations, if any lesions are observed. Ultrasound findings include hypoechoic lesions, thickened fibroadipose septa, and swelling of soft tissue. CT scan may reveal a hypodense or isodense mass, muscle swelling, disruption and injury of the surrounding tissues, periosteal reaction, and sequestration.^[2,3,6]^ On MRI, muscle infiltration demonstrates an increased signal intensity on T1-weighted images, intermediate signal intensity compared with fat on T2-weighted images, and homogenous diffuse enhancement when contrast material is used. This specific radiographic evidence assists in the differential diagnosis of muscle lymphoma.^[3,7]^ PET/CT is the preferred method for staging, treatment efficacy assessment, and possible extranodal disease detection or exclusion.^[6]^

Musculoskeletal invasion of lymphoma may become a differential diagnostic challenge. Entities with a similar clinical and radiographic presentation include sarcoma, metastatic carcinoma, melanoma, rhabdomyoblastoma, rhabdomyosarcoma, osteosarcoma, and purulent abscess. Histological evaluation of the lesion is the gold standard for definite diagnosis of the disease and further treatment approach selection.^[2–4]^ Approximately 66% of patients suffering a relapse have extranodal disease ^[8]^ and 50% of extranodal recurrences occur at previously uninvolved sites.^[9]^ In this context, a histopathologic assessment of the lesion is considered necessary. The backbone of DLBCL treatment is R-CHOP immunochemotherapy. The regimen is used for early stage as well as disseminated disease. In the first case, it may be combined with regional radiotherapy.^[1]^ Approximately 50% of the patients will relapse after treatment or may not respond to initial treatment. The treatment approach is then based on the goal, curative or palliative. Patients who can tolerate high-dose chemotherapy and autologous stem cell transplantation are started on a second-line salvage chemotherapy regimen. Palliative treatment may include chemotherapy with vincristine, cytarabine, alkylating agents, or anthracyclines, as well as local radiotherapy.^[1]^ The use of gemcitabine and vinorelbine in the setting of relapsed DLBCL is supported by 2 phase 2 trials (one of which combined the drugs with prednisone). The regimen is considered well-tolerated and efficient.^[10,11]^

Our case describes a primary nodal lymphoma, which at recurrence presented as an extranodal lymphoma. The disease had no initial extranodal involvement, was staged and treated as a nodal lymphoma, and responded well to treatment. After a disease-free survival interval of 3 months, the disease recurred at a single extranodal site, involving skeletal muscles. The remarkable element lies in the reappearance of the lymphoma at the left thigh muscles, with no radiographic or clinical evidence of involvement of lymph nodes, despite the extensive lymph node disease at initial presentation.

Although many cases of primary muscle lymphoma have been reported, 3 similar cases of isolated skeletal muscle recurrence have been described in the literature. A case of a paranasal sinus lymphoma recurrence, after initial complete response to treatment, at the gastrocnemius and soleus muscles was treated with cyclophosphamide, vincristine, prednisolone, and involved field radiotherapy.^[12]^ A recurrence of pancreatic DLBCL with involvement of the right masticator space and the left psoas muscle has been described, without other evidence of disseminated disease. The patient was treated with chlorambucil, procarbazine, prednisolone, vinblastine (CLLVPP) and prednisolone, doxorubicin, bleomycin, vincristine, etoposide (PABLOE), resulting into symptom improvement. Five months later, the patient died from meningeal lymphomatous involvement.^[13]^ The third case involves the recurrence of large cell lymphoma at the right psoas muscle after an initial complete remission of the disease. Three months after the diagnosis of the muscle relapse, the patient had abdominal surgery (debulking and right hemicolecomy) due to extensive disease progression in the abdomen. Second-line chemotherapy with VP16, methylprednisolone, arabinoside-C, and cisplatinum was administered.^[14]^

The further management of such recurrences remains to be clarified, as the odd biological behavior of the malignant cells dictates a special handling of the disease. The cellular and molecular characteristics of the neoplastic population in these cases must be studied, in order to provide prognostic and therapeutic evidence for future patients.
